# Minimum intensity projection of embossed quadrant-detection images for improved photoreceptor mosaic visualisation

**DOI:** 10.3389/fopht.2024.1349297

**Published:** 2024-03-13

**Authors:** Angelos Kalitzeos, Michel Michaelides, Alfredo Dubra

**Affiliations:** ^1^ Institute of Ophthalmology, University College London, London, United Kingdom; ^2^ National Institute for Health and Care Research, Biomedical Research Centre, Moorfields Eye Hospital, London, United Kingdom; ^3^ Byers Eye Institute, Stanford University, Palo Alto, CA, United States

**Keywords:** adaptive optics, quadrant-detection, non-confocal, photoreceptors, split-detection, minimum intensity projection, contrast

## Abstract

Non-confocal split-detection imaging reveals the cone photoreceptor inner segment mosaic in a plethora of retinal conditions, with the potential of providing insight to ageing, disease, and response to treatment processes, *in vivo*, and allows the screening of candidates for cell rescue therapies. This imaging modality complements confocal reflectance adaptive optics scanning light ophthalmoscopy, which relies on the waveguiding properties of cones, as well as their orientation toward the pupil. Split-detection contrast, however, is directional, with each cone inner segment appearing as opposite dark and bright semicircles, presenting a challenge for either manual or automated cell identification. Quadrant-detection imaging, an evolution of split detection, could be used to generate images without directional dependence. Here, we demonstrate how the embossed-filtered quadrant-detection images, originally proposed by Migacz et al. for visualising hyalocytes, can also be used to generate photoreceptor mosaic images with better and non-directional contrast for improved visualisation. As a surrogate of visualisation improvement between legacy split-detection images and the images resulting from the method described herein, we provide preliminary results of simple image processing routines that may enable the automated identification of generic image features, as opposed to complex algorithms developed specifically for photoreceptor identification, in pathological retinas.

## 1 Introduction

Adaptive optics scanning light ophthalmoscopy (AOSLO) affords the transverse resolution necessary to visualise the cone and rod photoreceptor mosaics in the living human eye, as well as a multitude of retinal features and pathologies (1, 2). Aging and disease affect these cell populations that are essential for vision, and therefore the ability to reliably quantify them is paramount for early changes to be captured, more sensitive longitudinal monitoring, and potentially become more precise endpoints in future gene therapies targeting these cells. Ultimately, complementary AOSLO modalities reflectance confocal and non-confocal split-detection are inherently limited to resolving cells affected in advanced disease and/or challenging eyes. Confocal imaging despite resolving the smallest of cells provides very low (if any) contrast in disease, and the bright semicircle of split-detection against its grey background is also of low contrast. Moreover, split-detection images contain a non-homogeneous, low spatial frequency signal thought to be originating from the retinal pigment epithelium. These challenges are translated to either time-consuming, error-prone manual annotation of photoreceptor mosaics or the use of highly sophisticated algorithms previously trained by the gold standard technique, which is manual annotation. 

The main goal of this paper is to demonstrate the application of a previously described image processing method shown for non-confocal quadrant-detection imaging on a new target, namely, photoreceptor cells. The motivation for this work is to ultimately address the inherent limitations of AOSLO and improve the visualisation of the photoreceptor mosaic. To this end, we present preliminary results from four representative conditions affecting these cells that may potentially lead to reducing the complexity of cell identification.

## 2 Methods

*En face* image sequences of photoreceptor mosaics were recorded using a custom-built AOSLO using light from a 790-nm super-luminescent diode (SLD; Superlum, Ireland). Confocal reflectance imaging revealed photoreceptor outer segments, whereas non-confocal multiple-scattered light revealed photoreceptor inner segments using an off-the-shelf fibre bundle quadrant-detection setup (BF42LS01, Thorlabs, Germany). Wavefront sensing was achieved using light from an 850-nm Superlum SLD, with the wavefront correction performed with a continuous sheet deformable mirror (DM97-15, ALPAO, France). Participants’ pupils were dilated using a drop of 1% tropicamide and 2.5% phenylephrine, each. A bite-bar provided head stability during imaging while fixating on a crosshair for around 9 s at a time, at each retinal location. Overlapping retinal locations were recorded so these could later be montaged either manually or automatically (3) into larger areas. Participants’ axial length was obtained to lateral-scale AOSLO images (IOLMaster 700, Carl Zeiss Meditec, Germany). Image distortion due to the use of a resonant scanner was removed by recording a grid of horizontal and vertical lines of known spacing (Ronchi ruling). Reference frames with minimal distortion due to eye movement were selected from each image sequence either manually or automatically (4), for registration and averaging of at least 40 images at each retinal location to increase the signal-to-noise ratio (5). Intraframe motion correction (de-warping) was then applied to remove any residual distortion (6).

**Figure 1:**
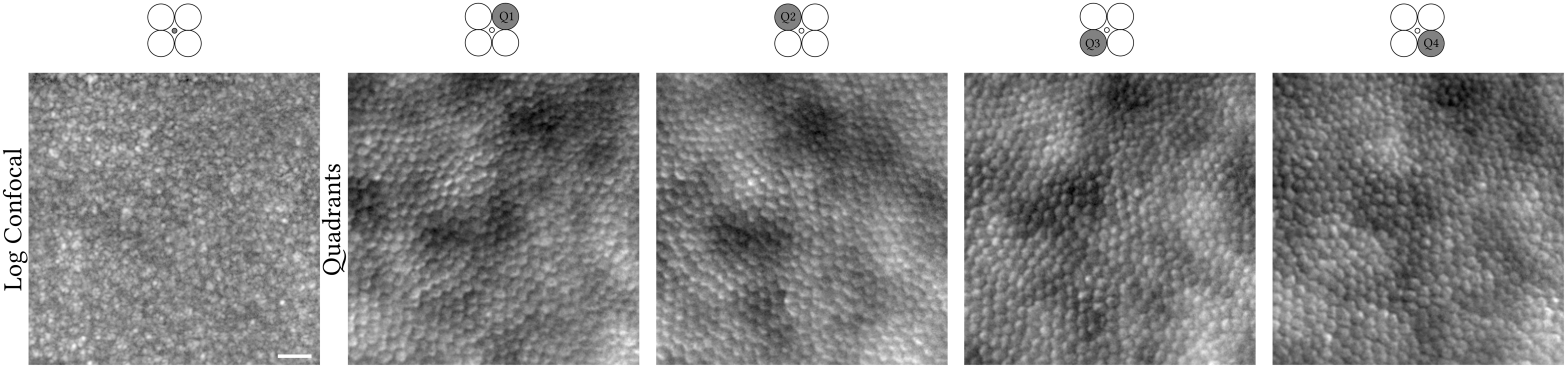
Confocal (logarithmic greyscale) and non-confocal quadrant-detection (Q1–Q4) images recorded simultaneously from the right eye of a 31-year-old X-linked retinitis pigmentosa patient. Light contributing to the creation of each image is collected from the confocal pinhole or fibre optic highlighted in dark grey, respectively. The centre of this photoreceptor mosaic is 226 microns away from the fovea. Scale bar is 20 microns.

**Figure 2:**
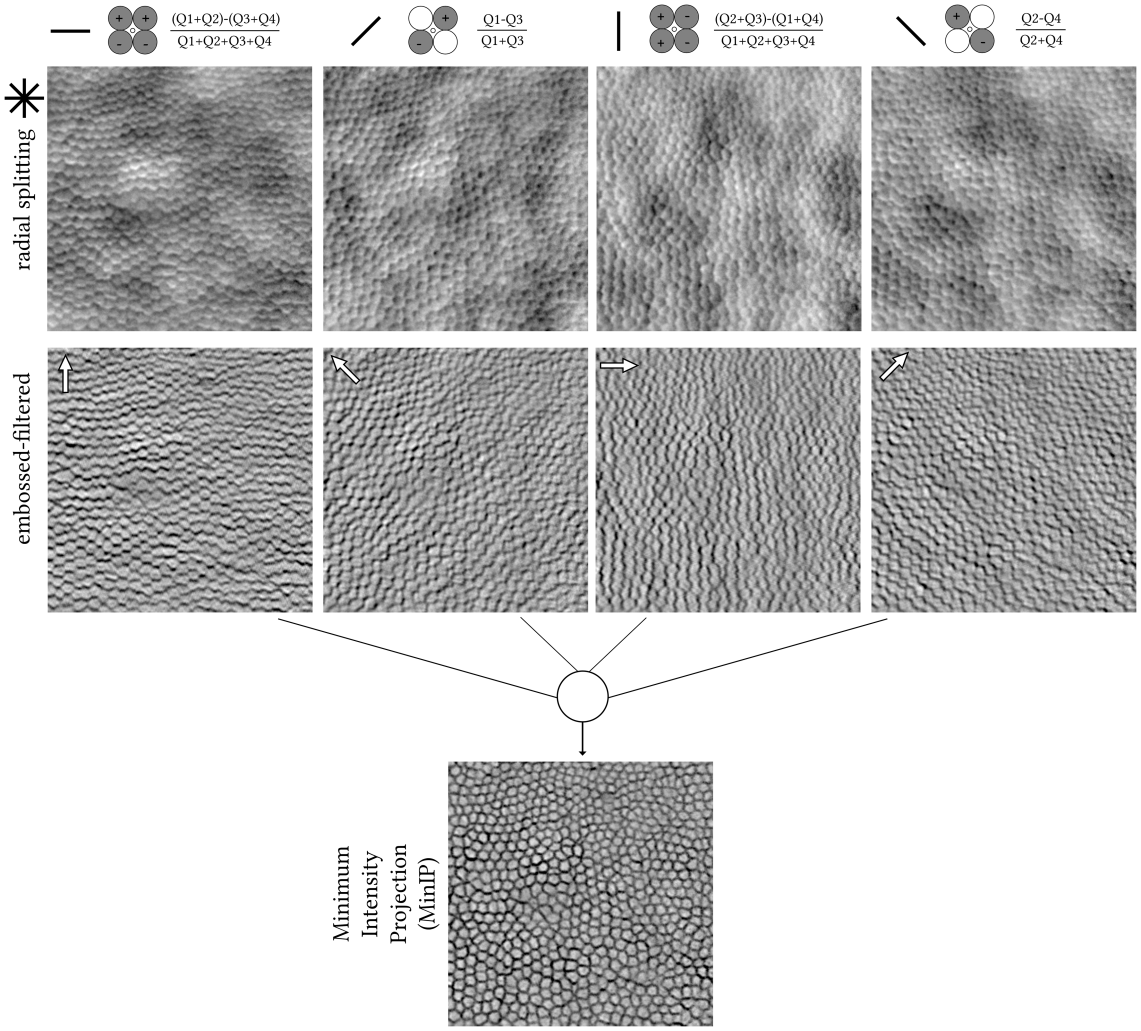
Pixel-by-pixel calculations from the raw quadrant-detection images shown in **Figure 1**. The angle of each of the four split axes is denoted with a solid black line. Radial splitting resulting images (top row); horizontal, 45°, vertical, and 135° from left to right, respectively. Photoshop-created embossed-filtered images using angles orthogonal to each split axis, with a direction from bright to dark cell borders (white arrows, middle row). Stack of four embossed-filtered images combined into one using Minimum Intensity Projection (MinIP, bottom row) in ImageJ. The centre of this photoreceptor mosaic is 226 microns away from the fovea.

The confocal pinhole was created by a tilted custom reflective binary mask that transmits light over a circular area 0.76 Airy disk in diameter (ADD; 37.5 μm) (**Figure 1**, column 1). The non-confocal light reflected by this mask was relayed using an achromatic doublet pair onto an 1-to-4 fan-out fibre optic bundle in a round configuration (**Figure 1**, columns 2–5). Although distinct from a previous approach (7), the current setup represents a close approximation and is conceptually the same, as each fibre captures light from a different quadrant (Q1–Q4) while providing a more mechanically stable approach than dividing non-confocal light into four quadrants using mirrors. Each of the four fibres has a 200-μm core diameter and a numerical aperture of 0.39. Their centres are 118.75 μm offset from the centre of the confocal pinhole. The image sequences of these four quadrants were mathematically combined similarly to that of split-detection, i.e., “legacy” vertical (90°) direction (8), whereas we also calculated the horizontal (0°), 45°, and 135° quadrant-detection images, which is afforded by dividing light into four rather than two detectors (**Figure 2**, black solid lines).The detection implementation is described in detail elsewhere (7). Photo-multiplier tube control voltages were adjusted by the operator throughout acquisition to maintain balanced mean pixel values across all quadrant detectors.

The quadrant-detection images were further processed using emboss filtering in Photoshop CS6 Extended (Adobe Inc.), as previously described (9). The filter parameters were empirically set to similar values as per the aforementioned publication irrespective of retinal condition or eccentricity, i.e., height of 5 pixels, amount of 150%, and angles orthogonal to each radial split axis and in the direction toward the dark cell border (**Figure 2**, white arrows). Finally, from each stack of four embossed images, a single final image was generated using ImageJ’s (10) minimum intensity Z-project, hereon, MinIP (**Figure 2**).

A Photoshop actions script to automate the creation of the embossed-filtered images as well as the ImageJ macro to batch process the resulting images into the single final MinIP image is provided (**Supplementary Material**).

**Figure 3:**
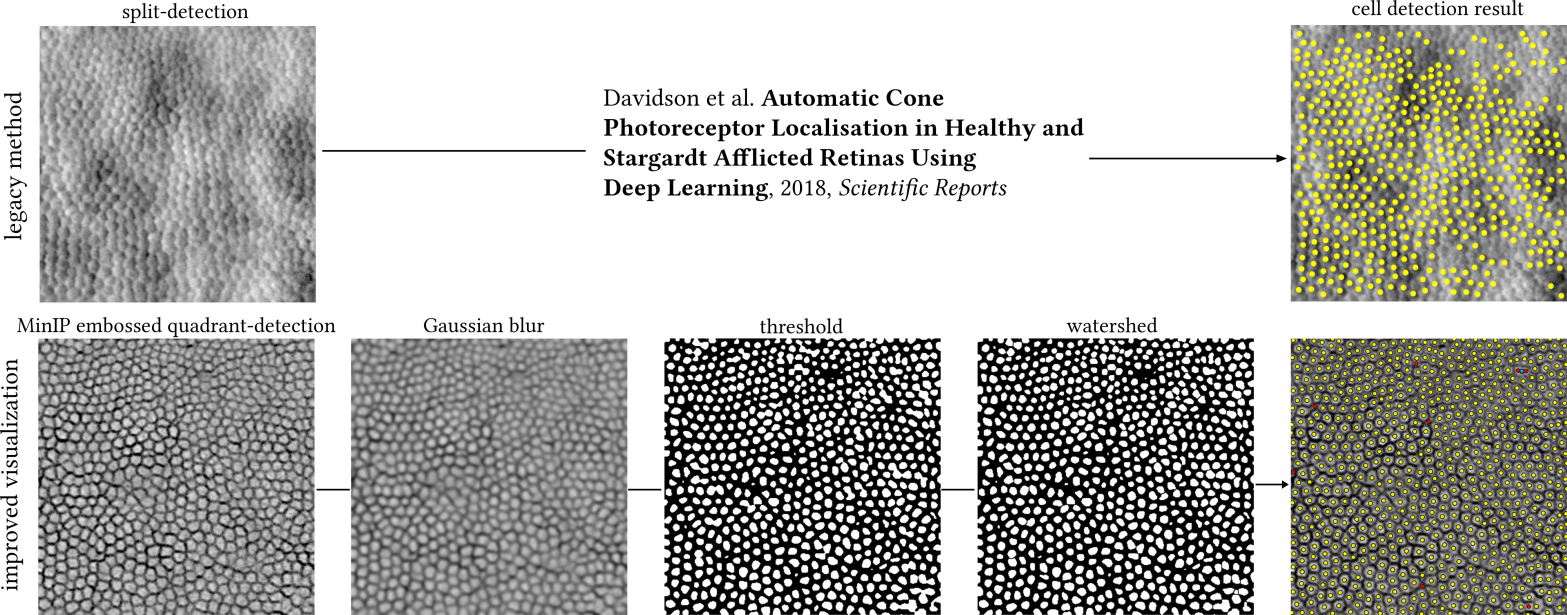
Top row: legacy split-detection image (as defined in **Figure 2**, column 3) segmented by a previously published Deep Learning method. Bottom row: cell segmentation of the MinIP embossed quadrant-detection image shown in **Figure 2** (400 pixels square) using ImageJ. Image processing steps could be run as a single macro, here shown individually for illustration purposes. MinIP; Minimum Intensity Projection. Yellow dots indicate overlap between automated cell identification (ImageJ) and manual annotation, red dots indicate cells manually annotated missed by ImageJ (false negatives), and cyan dot indicates two cells mislabelled as one. The centre of this photoreceptor mosaic is 226 microns away from the fovea.

To explore whether the resulting MinIP images represent a visualisation improvement of the photoreceptor mosaic over their equivalent legacy split-detection images in terms of automated cell identification, we performed simple image processing routines using ImageJ (version 1.54g) as a demonstrative example. First is by applying a Gaussian blur with a radius value of 2 pixels, binarizing the image using thresholding, and then applying watershed separation for neighbouring cells not to be falsely merged into one. Cutoff values for thresholding were chosen guided by the histogram plots of each image at the trough between the two peaks. Lastly, photoreceptor cells were automatically identified using the “Find Maxima” function, which returns individual cell centroids, as shown in **Figure 3**. The equivalent legacy vertical split-detection image fed to a Machine Learning algorithm previously published (11) can be seen in the same figure for direct comparison.

**Figure 4:**
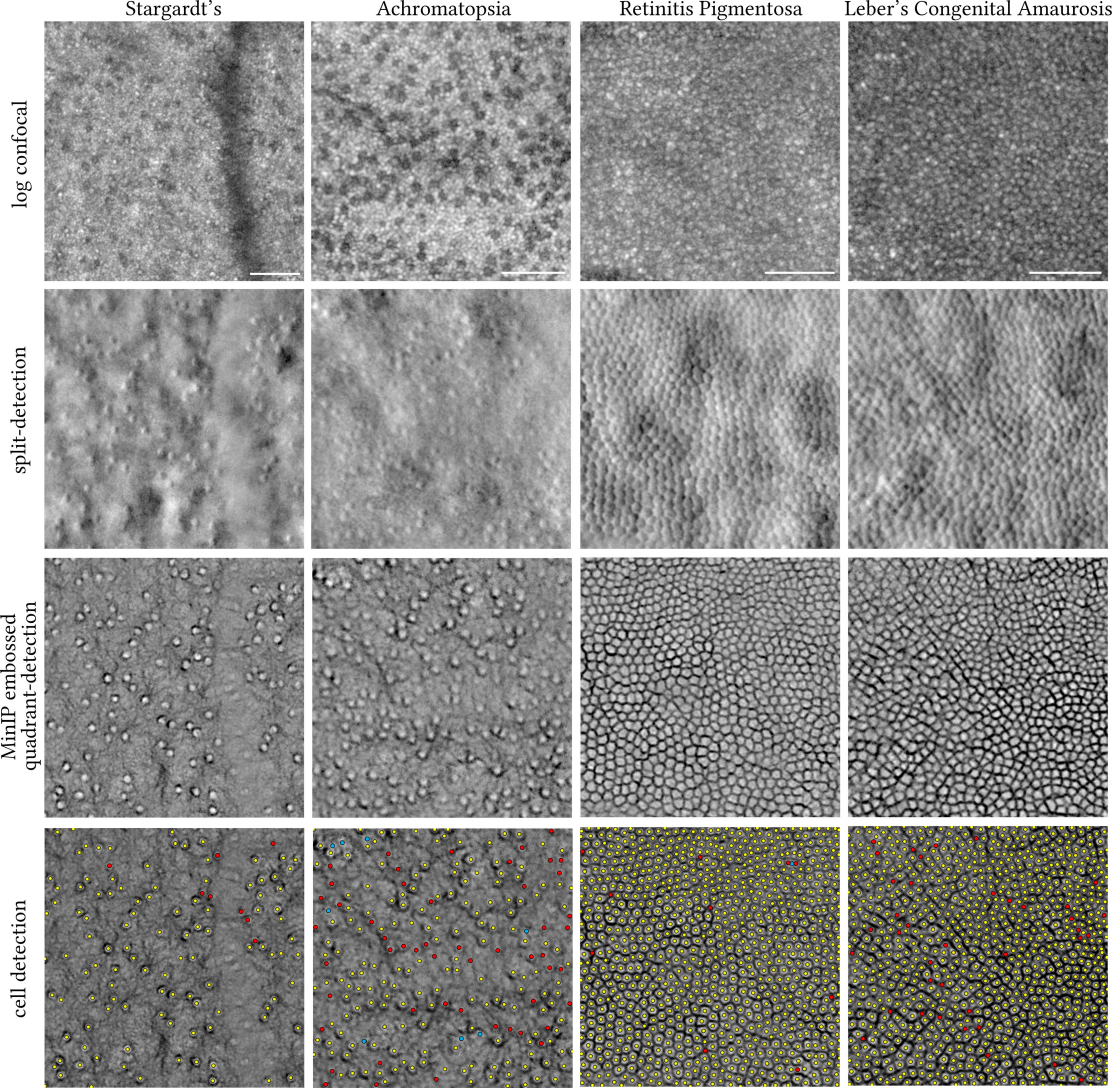
Visual comparison of legacy split-detection (as defined in **Figure 2**, column 3) over MinIP embossed quadrant-detection in Stargardt’s (16 years old), achromatopsia (38 years old), retinitis pigmentosa (31 years old), and Leber’s congenital amaurosis type 2 (16 years old) (430 pixels square each). Top row shows the equivalent confocal images in logarithmic greyscale. Bottom row shows the results of cell detection using ImageJ. Only cone photoreceptors were targeted in Stargardt’s and achromatopsia images. Yellow dots indicate overlap between automated cell identification (ImageJ) and manual annotation, red dots indicate cells manually annotated missed by ImageJ (false negatives), and cyan dots indicate either clusters of rods misidentified as cones by ImageJ (false positives) for the achromatopsia image or two cells mislabelled as one for the retinitis pigmentosa image. MinIP; Minimum Intensity Projection. The centre of each square crop is 2.7, 0.6, 0.2, and 0.15 mm away from the fovea, from left to right, respectively. Scale bars are 50 microns.

Last, depending on the condition, the Stargardt’s and achromatopsia non-confocal images could only resolve cone photoreceptors, whereas the retinitis pigmentosa and Leber’s congenital amaurosis type 2 non-confocal images may contain both cone and rod photoreceptors (**Figure 4**). To establish the ground truth for these representative conditions directly affecting cone and/or rod photoreceptors, all centroids of applicable cell types were manually annotated by a single grader (AK) and compared against the automatically identified ones using the steps described in **Figure 3** for the MinIP embossed images.

## 3 Results

The application of the Migacz et al. method in photoreceptor mosaics is illustrated in **Figures 1**, **2** by example of a parafoveal crop from an adult retinitis pigmentosa retina (400 pixels square) (12). The embossed-filtered images (intermediate step) shown in **Figure 2** are devoid of the low spatial frequency content of the input images, stemming from back-scattering of the retinal pigment epithelium and/or choroid, similarly for the fusion of the four images into the final MinIP embossed quadrant-detection image. Qualitatively, it is evident that some cells’ borders are more pronounced than others.

Manual annotation of the centre of every cell (in this example, either cone or rod) resulted in a total of 752 cells, whereas the brief exploration of simple image processing routines to automate such annotation missed eight cells (false negatives, red dots, 1%) of which two were mislabelled as one (cyan dot). The more complex, state-of-the-art method missed 260 (34.6% of total) cells on the equivalent legacy split-detection image (**Figure 3**).

We also present the results of the embossed quadrant-detection MinIP method in representative images (430 pixels square, each) of retinae whose photoreceptors are affected, namely, Stargardt’s disease, achromatopsia, and Leber’s congenital amaurosis type 2 (LCA2) (**Figure 4**). Thresholding using ImageJ as described in **Figure 3** was also applied to these images, and resulting cell annotations (manual and automatic) are shown. For the Stargardt’s image, 117 cones were manually annotated of which eight were missed by thresholding (red dots, false negatives, 6.8%). For the achromatopsia image, 221 cones were manually annotated (aided by confocal reflectance) of which 52 were missed by thresholding (red dots, false negatives, 23.5%) and eight clusters of rods were falsely marked as cones (cyan dots, false positives, 3.6%). For the (slightly enlarged in area than **Figure 3**) retinitis pigmentosa image, 867 cells were manually annotated of which 10 cells were missed by thresholding (red dots, false negatives, 1.1%) of which two were mislabelled as one (cyan dot). For the LCA2 image, 914 cells were manually annotated of which 38 were missed by thresholding (red dots, false negatives, 4.1%).

## 4 Discussion

The methodology described here was previously applied to the retinal vasculature (9) and—slightly modified—to vitreous cortex hyalocytes (12). Here, we apply it to retinal photoreceptor mosaics in a range of conditions affecting these cells and provide the software (Photoshop actions and ImageJ macro) used to implement and automate the creation of MinIP embossed quadrant-detection images. While not a direct goal of this work, we briefly explored the feasibility of performing simple image processing routines using ubiquitous, open-source software to deliver automated cell identification in non-confocal AOSLO MinIP embossed quadrant-detection images.

This is not the first study to describe a radial splitting detection scheme with the goal to increase contrast for retinal cells (13–15). However, here we employ simultaneous acquisition of confocal and non-confocal images rather than in a sequential fashion by means of a commercially available fibre optic bundle and keep the number of non-confocal channels to the minimum (four), attempting to strike a balance between improved cell border visualisation and simplicity to implement and maintain. Importantly, this does not increase the time needed to acquire the images and it does not require additional spatial image registration, maintaining temporal registration.

The resulting images of photoreceptors described here are derivatives of (multiple axes of) split-detection and as such are governed by the same limitations in terms of resolving the smallest of cells, either foveal cones or rods. Reflectance confocal AOSLO would be the modality of choice for such cells. An exception to this rule is in the case of degeneration/disruption where cells are less numerous and thus larger in size. Other limitations of this work include the lack of direct comparison between images from a legacy split-detection AOSLO and the fibre-optic bundle quadrant-detection AOSLO described here, on the same retinae. The assumption that the images are of comparable quality would need to be tested to exclude a potential image quality bias depending on AOSLO hardware used. The methods applied and images shown here were processed retrospectively from past natural history studies and were randomly chosen to represent each condition without *a priori* knowledge of the quality of the MinIP embossed images. However, it would be beneficial to apply the method prospectively in larger cohorts in both unaffected and affected retinae to explore its applicability further. To illustrate the effect of MinIP embossed filtering across various retinal eccentricities for all four conditions, the full montages are provided from which the images shown in **Figure 4** were cropped from (**Supplementary Figures 1–4**).

Another consideration that we did not account for in this work are the potential differences in photoreceptor inner segment shape (and thus, size) between the well-described, legacy split detection images and the current MinIP embossed images. Future work could quantitatively compare such differences by means of previously described software (16) and the use of (for example) ImageJ (“Analyze Particles”) for the type of images presented herein.

Split-detection works on the principle of directionality by design; non-confocal light is split along the vertical axis, and the contrast created to resolve inner segments manifests as a dark and a bright semicircle to the right and left borders of any given cell. However, target structures that are oriented along the split-detection axis would not be resolved or, if they are oriented diagonally, they would be faintly or partially resolved. When it comes to retinal photoreceptor degeneration, not all cells are uniformly circular or symmetrical along a fixed axis. This is the reason why this work here focuses entirely on affected retinae, which are the most challenging to resolve and thus quantify. Additionally, the bright border of each cell provides low contrast against the non-uniform grey background. Quadrant-detection essentially builds upon split-detection by increasing the number of axes from one to a total of four in a radial splitting geometry. This provides several advantages when it comes to visualising photoreceptor cells: directionality is not crucial anymore to establish the necessary contrast; even imperfect image focusing yields satisfactory cell border contrast, as can be seen in the top left corner of the image from the LCA2 patient.

As a direct consequence of the above, advantages may also be brought forward to the cell identification domain. Historically, it has been both challenging and computationally complex to automatically annotate split-detection images of the photoreceptor mosaic (11, 16–19). Albeit the primary goal of this work is not to improve cell identification but rather to reduce its complexity, we briefly explore how simple image processing routines with no specialist software may provide an indirect way to showcase the improved visualisation of cell borders and put the work here into context with one of the previously published methods for automatic photoreceptor localisation. Even though the two techniques shown in **Figure 3** are not directly comparable due to the fact that the legacy one was trained on images from Stargardt’s patients and the current methods’ example used belongs to a retinitis pigmentosa patient, the salient point is that the MinIP embossed quadrant-detection image required no pretrained model. Manual annotation of cells may always be the gold standard when it comes to cell quantification, but the work presented here could be a step closer to shorter research staff training times and to abolishing the need for multiple graders due to the improved visualisation of cell borders. Postprocessed MinIP embossed quadrant-detection AOSLO images resemble those of *ex vivo* microscopy images of cells with well-defined borders against a uniform background without the low spatial frequency features in the source images. As a consequence, simpler and more widely used image processing pieces of software (20) are bound to facilitate their broader adoption from the research community.

## Data availability statement

The datasets presented in this study can be found in online repositories. The names of the repository/repositories and accession number(s) can be found below: https://www.frontiersin.org/articles/10.3389/fopht.2024.1349297/abstract#supplementary-material.

## Ethics statement

The studies involving humans were approved by London - Camden & Kings Cross Research Ethics Committee. The studies were conducted in accordance with the local legislation and institutional requirements. The participants provided their written informed consent to participate in this study.

## Author contributions

AK: Conceptualisation, Data curation, Formal analysis, Investigation, Methodology, Software, Visualisation, Writing – original draft, Writing – review & editing. MM: Funding acquisition, Supervision, Validation, Writing – review & editing. AD: Conceptualisation, Funding acquisition, Investigation, Resources, Supervision, Validation, Visualisation, Writing – review & editing.

## Supplementary material

Supplementary Figures 1-4 Full montages of the photoreceptor mosaics for all four conditions shown in . White asterisks indicate the foveal centre. White rectangles on the inset, macroscopic images indicate the extent of the area imaged with Adaptive Optics Scanning Light Ophthalmoscopy. Montages comprise minimum intensity projection (MinIP) of embossed quadrant-detection images for Stargardt’s, achromatopsia, retinitis pigmentosa and Leber’s congenital amaurosis type 2, respectively.

## References

[1] Scoles D Higgins BP Cooper RF Dubis AM Summerfelt P Weinberg DV . Microscopic inner retinal hyper-reflective phenotypes in retinal and neurologic disease. Invest Ophthalmol Vis Sci. (2014) 55:4015–29. doi: 10.1167/iovs.14-14668 PMC4078949 24894394

[2] Morgan JIW Chui TYP Grieve K . Twenty-five years of clinical applications using adaptive optics ophthalmoscopy [Invited]. Biomed Optics Express. (2023) 14:387. doi: 10.1364/BOE.472274 PMC9841996 36698659

[3] Davidson B Kalitzeos A Carroll J Dubra A Ourselin S Michaelides M . Fast adaptive optics scanning light ophthalmoscope retinal montaging. Biomed Opt Express. (2018) 9:4317–28. doi: 10.1364/BOE.9.004317 PMC6157757 30615701

[4] Salmon AE Cooper RF Langlo CS Baghaie A Dubra A Carroll J . An automated reference frame selection (ARFS) algorithm for cone imaging with adaptive optics scanning light ophthalmoscopy. Trans Vision Sci Technol. (2017) 6:9. doi: 10.1167/tvst.6.2.9 PMC5381332 28392976

[5] Dubra A Harvey Z . Registration of 2D images from fast scanning ophthalmic instruments. In: Biomedical Image Registration: 4th International Workshop, WBIR 2010. Lübeck, Germany: SpringerLink (2010). 10.1007/978-3-642-14366-3_6 PMC13282932 42328045

[6] Salmon AE Cooper RF Chen M Higgins B Cava JA Chen N . Automated image processing pipeline for adaptive optics scanning light ophthalmoscopy. Biomed Optics Express. (2021) 12:3142. doi: 10.1364/BOE.418079 PMC8221964 34221651

[7] Sredar N Razeen M Kowalski B Carroll J Dubra A . Comparison of confocal and non-confocal split-detection cone photoreceptor imaging. Biomed Opt Express. (2021) 12:737–55. doi: 10.1364/BOE.403907 PMC7901313 33680539

[8] Scoles D Sulai YN Langlo CS Fishman GA Curcio CA Carroll J . *In vivo* imaging of human cone photoreceptor inner segments. Invest Ophthalmol Vis Sci. (2014) 55:4244–51. doi: 10.1167/iovs.14-14542 PMC4095721 24906859

[9] Pinhas A Migacz JV Zhou DB Castanos Toral MV Otero-Marquez O Israel S . Insights into Sickle Cell Disease through the Retinal Microvasculature: Adaptive Optics Scanning Light Ophthalmoscopy Correlates of Clinical OCT Angiography. Ophthalmol Sci. (2022) 2:100196. doi: 10.1016/j.xops.2022.100196 36531581 PMC9754983

[10] Schneider CA Rasband WS Eliceiri KW . NIH Image to ImageJ: 25 years of image analysis. Nat Methods. (2012) 9:671–5. doi: 10.1038/nmeth.2089 PMC5554542 22930834

[11] Davidson B Kalitzeos A Carroll J Dubra A Ourselin S Michaelides M . Automatic cone photoreceptor localisation in healthy and stargardt afflicted retinas using deep learning. Sci Rep. (2018) 8:7911. doi: 10.1038/s41598-018-26350-3 29784939 PMC5962538

[12] Migacz JV Otero-Marquez O Zhou R Rickford K Murillo B Zhou DB . Imaging of vitreous cortex hyalocyte dynamics using non-confocal quadrant-detection adaptive optics scanning light ophthalmoscopy in human subjects. Biomed Opt Express. (2022) 13:1755–73. doi: 10.1364/BOE.449417 PMC8973177 35414987

[13] Rossi EA Granger CE Sharma R Yang Q Saito K Schwarz C . Imaging individual neurons in the retinal ganglion cell layer of the living eye. Proc Natl Acad Sci. (2017) 114:586–91. doi: 10.1073/pnas.1613445114 PMC5255596 28049835

[14] Mecê P Gofas-Salas E Rui Y Zhang M Sahel J-A Rossi EA . Spatial-frequency-based image reconstruction to improve image contrast in multi-offset adaptive optics ophthalmoscopy. Optics Lett. (2021) 46:1085–8. doi: 10.1364/OL.417903 PMC9202470 33649663

[15] Gofas-Salas E Rui Y Mecê P Zhang M Snyder VC Vienola KV . Design of a radial multi-offset detection pattern for in *vivo* phase contrast imaging of the inner retina in humans. Biomed Optics Express. (2022) 13:117–32. doi: 10.1364/BOE.441808 PMC8803027 35154858

[16] Liu J Jung H Dubra A Tam J . Cone photoreceptor cell segmentation and diameter measurement on adaptive optics images using circularly constrained active contour model. Invest Ophthalmol Visual Sci. (2018) 59:4639. doi: 10.1167/iovs.18-24734 30372733 PMC6154284

[17] Cunefare D Cooper RF Higgins B Katz DF Dubra A Carroll J . Automatic detection of cone photoreceptors in split detector adaptive optics scanning light ophthalmoscope images. Biomed Opt Express. (2016) 7:2036–50. doi: 10.1364/BOE.7.002036 PMC4871101 27231641

[18] Liu J Jung H Dubra A Tam J . Automated photoreceptor cell identification on nonconfocal adaptive optics images using multiscale circular voting. Invest Ophthalmol Visual Sci. (2017) 58:4477. doi: 10.1167/iovs.16-21003 28873173 PMC5586244

[19] Cunefare D Langlo CS Patterson EJ Blau S Dubra A Carroll J . Deep learning based detection of cone photoreceptors with multimodal adaptive optics scanning light ophthalmoscope images of achromatopsia. Biomed Opt Express. (2018) 9:3740. doi: 10.1364/boe.9.003740 30338152 PMC6191607

[20] Stringer C Wang T Michaelos M Pachitariu M . Cellpose: A generalist algorithm for cellular segmentation. Nat Methods. (2021) 18:1. doi: 10.1038/s41592-020-01018-x 33318659

